# Incipient Social Groups: An Analysis via In-Vivo Behavioral Tracking

**DOI:** 10.1371/journal.pone.0149880

**Published:** 2016-03-23

**Authors:** Jamin Halberstadt, Joshua Conrad Jackson, David Bilkey, Jonathan Jong, Harvey Whitehouse, Craig McNaughton, Stefanie Zollmann

**Affiliations:** 1 Department of Psychology, University of Otago, Dunedin, New Zealand; 2 Department of Psychology, University of Maryland, College Park, Maryland, United States of America; 3 Coventry University, Coventry, United Kingdom; 4 Institute of Cognitive and Evolutionary Anthropology, University of Oxford, Oxford, United Kingdom; 5 Animation Research Limited, Dunedin, New Zealand; University of Goettingen, GERMANY

## Abstract

Social psychology is fundamentally the study of individuals in groups, yet there remain basic unanswered questions about group formation, structure, and change. We argue that the problem is methodological. Until recently, there was no way to track who was interacting with whom with anything approximating valid resolution and scale. In the current study we describe a new method that applies recent advances in image-based tracking to study incipient group formation and evolution with experimental precision and control. In this method, which we term “in vivo behavioral tracking,” we track individuals’ movements with a high definition video camera mounted atop a large field laboratory. We report results of an initial study that quantifies the composition, structure, and size of the incipient groups. We also apply in-vivo spatial tracking to study participants’ tendency to cooperate as a function of their embeddedness in those crowds. We find that participants form groups of seven on average, are more likely to approach others of similar attractiveness and (to a lesser extent) gender, and that participants’ gender and attractiveness are both associated with their proximity to the spatial center of groups (such that women and attractive individuals are more likely than men and unattractive individuals to end up in the center of their groups). Furthermore, participants’ proximity to others early in the study predicted the effort they exerted in a subsequent cooperative task, suggesting that submergence in a crowd may predict social loafing. We conclude that in vivo behavioral tracking is a uniquely powerful new tool for answering longstanding, fundamental questions about group dynamics.

## Introduction

Social psychology has been described as the study of how individuals behave in groups [1], which is a testament to the importance of grouping in our social lives. Whether it is joining a conversation circle at a party or taking a seat at a café, we are consistently decreasing the physical space between ourselves and chosen others as an overture of affiliation, and a precursor to forming more meaningful social units. Nevertheless, even as researchers propose, test, and debate multi-factorial theories of group formation [2, 3], we lack knowledge about some of the most fundamental features of group formation and composition. For example, we know that the size of groups is heavily dependent on social context [4, 5], but not what the “baseline” size of an emerging group is. We know that a person’s visibility in a group relates to his or her perceived influence [6, 7], but not what predicts, or is predicted by such visibility. We know that groups vary in their density [8, 9], but not whether we can infer anything from how deeply people are embedded in them. In this paper, we use a new method of quantifying group formation–“in vivo behavioral tracking”–to observe how superficial traits shape “incipient social groups,” the first clusters that form among indistinct crowds, and the gateway to more intimate social behavior.

### Incipient social groups as affiliative structures

The mechanism of group formation—physical proximity—is at the heart of any investigation into human social behavior, because any convergence of individuals is typically nonrandom and psychologically significant. The classic literature on “personal space” delineates a proximal boundary into which only close others are permitted [10], such that approaching that boundary signals increasing psychological intimacy. The initiation of social contact maps closely onto entry into others’ physical space [11, 12]. Thus, in a very real sense, physical proximity is not just a proxy for social contact; it *is* social contact, and as such can, when observed on a larger scale, give direct insight into emergent social ties.

A number of previous field studies have used physical proximity to predict self-identified group membership and shared beliefs and values. For example, Freeman and Webster [13] collected systematic field observations from a crowded beach over 31 days, in which they observed the spatial locations and interactions among the beachgoers. The authors found that spatial proximity over the 31-day interval not only predicted interaction frequency, but also participants’ inferences of psychological similarity: beachgoers over-estimated the degree that their neighbors (those who sat nearer to them on the beach) shared their beliefs and attitudes. Researchers studying the acquaintance process in student dormitory rooms have replicated this result [14, 15], showing that proximity predicts not only students’ social ties, but also plays a more complex role in students’ emergent social networks.

Even simulated models of group formation have used physical proximity as a measure of social grouping. Recently, Gray and colleagues extended previous models of cooperation [16, 17] to demonstrate that homogeneous computer agents move physically closer to others who reciprocate cooperative initiatives and are held in good favor by already-formed “group members” [18]. Over time, agents banded together into discrete proximal groupings, which related to their desire for future interaction with group members. These links between physical and psychological closeness, in combination with the fieldwork on personal space and proxemics [19, 20], suggest that humans do not approach each other arbitrarily. Rather, when people decide to share their physical space with others, they also commit to sharing their social identity.

### Exploring Group Characteristics

Given the significance of proximity for predicting and understanding how people will affiliate, it is surprising that more is not known about the parameters of incipient groups. At least part of this gap is methodological: there has been no way to keep track of who is approaching whom with anything approximating valid resolution, scale, and control. Because groups in the field do form spontaneously and unpredictably, and involve people whose characteristics and motivations are unknown, observing them, much less controlling them, is a challenge. Previous social psychology studies have often relied either on indirect or approximate indicators of proximity (e.g. rooms in a residence hall), or ecologically deficient laboratory paradigms that operationalize groups in terms of decision-making tendencies and attitudes (see [21]). The result has been an historical tradeoff between ecological validity and experimental control: researchers either sacrifice the spontaneity and freedom of movement of the field, or the control and precise measurement of the laboratory.

However, advances in technology have made it possible to resolve this tradeoff by tracking the movements, proximities, and identities of large groups of people with very high temporal and spatial resolution. In the current study we apply this technology in a new method of “in-vivo spatial tracking” to track sets of 40–50 strangers interacting in a 600m^2^ space. We use this technique to answer several basic questions about the first stages of social group formation: (1) Do people physically group based on shared physical traits? (2) Do these traits also predict the physical position of individuals in their groups? And (3) What does variability in social distance say about future prosocial behavior?

#### Social identity and group composition

When groups first emerge from a relatively indistinct whole, what individual characteristics determine what they look like? Although little research has examined the critical moment of physical group formation, a wealth of literature suggests that groups do not assemble randomly, but on the basis of social “fault lines.” According to Social Identity Theory, for example, individuals identify with and favor others who share common features, even arbitrary or superficial ones. For example, “minimal group” research shows a robust preference for others (e.g., in a laboratory resource allocation task) who supposedly share one’s preference for abstract art or one’s nametag color [22–24]. Analogously, participants in “bogus stranger” studies typically favor (fictional) others who share their attitudes and beliefs [25]. There have been fewer studies, however, on whether such biases translate into physical grouping. “Free-range data harvesting” has revealed that randomly sampled dyads show a range of physical and psychological similarities [21], and studies in the close relationships literature have found that partners are more similar in attractiveness [26–28], age [29, 30], ethnicity [31, 32], and invisible characteristics [33, 34] than expected by chance, but whether such traits also predict the composition of incipient physical groups is unknown. The current study directly examines whether strangers with shared physical characteristics are more likely than chance to physically cluster together; we focus specifically on gender and physical attractiveness, two superficial social traits with well-documented social homophily effects [35, 26–28] that vary appropriately in a largely Caucasian student sample. Furthermore, by modeling the strength of these matching effects over repeated interactions, we test for whether individuals coalesce into progressively more homogenous groups, or whether homophily breaks down as group members become more familiar with one another.

#### Who is the center of their group?

A fundamental dimension of incipient groups is their physical dynamic: where individuals position themselves relative to one another. As sets of individuals first calve off from a larger whole, physical position is likely to have a dramatic effect on the nature and effectiveness of the social group that ultimately evolves, since social information is extracted automatically from visual information alone. Taylor and Fiske [6], for example (see also [7]), famously documented the role of visual salience in attributions of causality: participants judged confederates as more influential when they had a clearer view of them. Therefore, people who, for whatever reason, find themselves at the spatial center of an emergent group may also find themselves its leader.

Although no empirical research in social psychology has studied the physical structure of incipient groups, Pennebaker’s theoretical work on “social physics” provides a useful metaphor [36, 37]. Pennebaker theorized that group formation is analogous to physical attraction and repulsion, such that social “attractors” exert psychological force that, like gravitational force, draw other people into their vicinity. Examples of social attractors, according to Pennebaker, are beer stands or food tables at student parties, which often lie at the center of social groups. Similarly, we propose that other *people* can serve as attractors, quantified as the average distance from a given individual to all others in the individual’s proximate group. Gender and physical attractiveness are also plausible candidates as attractor-enabling features. Perhaps as a function of short-term mating strategies, men show greater attraction to women in speed dating paradigms than vice versa [38], and individuals of both sexes show a halo effect based on physical attractiveness (see [39] for a meta-analysis). We hypothesized, therefore, that women and attractive individuals would be more likely found in the middle of their groups. We also considered age and minority status as possible attractors, as each has some precedence in the literature on social salience [40–43].

#### Social distance and social loafing

While our first two questions concern small groups that form out of larger wholes, our third question concerns the larger groups themselves and the inferences that can be drawn from their structure. There is no consensus on the “natural” density of crowds, even though there are known—and potentially competing—effects of others’ presence on behavior. According to Zajonc’s Drive Theory, the presence of other people can either make individuals feel aroused and self-aware, or relaxed and anonymous, depending on the size of the group and the transparency of individual performance [44, 45]. There is some evidence, for example, that larger groups are more productive and more effective problem solvers, [46, 47], but other evidence that larger groups are less cooperative than smaller groups [48, 49], and are more likely to suffer diffusion of responsibility [50, 51] and social loafing [52]. More recent research on teamwork has shown that individuals in larger groups over-claim responsibility [53], and underperform with respect to expectations [54] since they are able to escape responsibility behind relative anonymity. Indeed, a recent comprehensive review of group behavior has claimed that “Pathologies of groups (e.g. social loafing, depletion of resources/commons dilemmas, failure to pool information, groupthink) are linked to submerging the individual self in the group” [55].

Individuals’ physical “submergence” in a group can be operationalized as the mean distance between them and all other group members, where individuals with the lowest mean distance are the most embedded [56]. Our specific question was whether this measure of embeddedness would predict antisocial behavior at a later point in time, as measured by “social loafing” in a task requiring the whole group’s cooperation. To answer this question, we measured embeddedness as participants mingled at the beginning of the experiment and later measured cooperation as the speed with which participants collected tokens scattered across the experimental area, a classic social dilemma in which individuals’ goals (i.e., to minimize effort) are achieved at the expense of the group’s (to complete the task in a timely way). Our hypothesis was that individuals who embed themselves deeply in a group would also be more likely to loaf when that group is asked to work together. Such a relationship could either indicate that social loafers evade identification by submerging themselves in crowds or that crowd submergence primes a sense of anonymity that influences later social loafing.

#### Summary of the Present Research

In sum, the current study provides the first direct look at the size, composition, dynamics, and implications of incipient social grouping, the moment at which merely coincidental gatherings of human beings first crystallize into psychologically significant subsets. Using a new method of spatial tracking, we test three hypotheses that have been foreshadowed by previous literature, but never directly tested. First, we predict that physical groups form on the basis of superficial characteristics (in this case, gender and attractiveness). Second, we predict that attractive individuals and women are especially likely to be positioned in the center of their groups. And third, we hypothesize that crowd embeddedness can significantly predict people’s tendency to socially loaf.

## Method

### Participants

One hundred and seventy-two students (41 men, 130 women, 1 “other,” not included in the gender analyses; mean age = 21.43, SD = 4.42; 114 white/ethnic majority, 58 non-white/ethnic minority) volunteered in exchange for NZ$40 to cover their travel expenses. Participants were run in one of four sessions, which took place back-to-back over a single day in the Forsyth-Barr Stadium in Dunedin, New Zealand. The study as a whole was described to participants as “an exploration of the viability of using [the Forsyth-Barr Stadium] for social science research.” Participants were aware that the study might be filmed, but not that their movements would be tracked. No participant indicated any concern about, or awareness of, the mounted camera.

### Ethics Statement

This study was approved by the ethics committee at the University of Otago. All participants provided written informed consent to participate in the study.

### In-vivo behavioral tracking

Our method draws in part from the growing literature in animal behavior that employs real-time tracking to gather data on animals and insects as they forage [57], hunt [57, 58], mate [59], socially interact [60], and react to sensory stimuli [61]. Much of this research employs automated image-based tracking, wherein data on movement and position are derived from videos ([62, 63], see [64] for a review). Some studies have adapted these image-tracking paradigms to examine human behavior (e.g., eye gazing or crowd structure [65–67]) but most human research has used “social sensors” or “sociometric badges” for example to track organizational behavior ([68, 69], see [70] for a review), face-to-face interactions during coffee breaks [71], and gender differences in cooperation [72]. However, sociometric badges also feature significant noise (with accuracy ranging from 1 to 3 meters in previously published research; [72, 73]). This range of error allows for the detection of probable social interactions (with some risk of false positives and negatives ([72], p8) but does not allow for fine-grained analysis of internal group dynamics. Furthermore, sociometric badges involve the significant disadvantage that wearing the badges can create reactance or experimenter demand effects [70].

These issues aside, we also note that both sociometric studies and image-based tracking studies have previously been conducted in observational settings, such as the workplace [68–70] or a crowded street [65]. Observational tracking has advantages; the method allows large groups of people to approach and avoid each other without significant environmental constraint or experimenter interference. However, observational methods also sacrifice important experimenter control, such as the ability to manipulate participants’ social context and interaction partners. Our paradigm, which combines the strengths of previous research, is achieved with a high-resolution camera mounted far and directly above the individuals interacting in an indoor stadium, in conjunction with custom-built software that can identify and plot individuals’ location in two-dimensional space for any foreseeable group size, length and type of interaction, proximity, and time scale.

### Experimental Procedure

**Fig 1:**
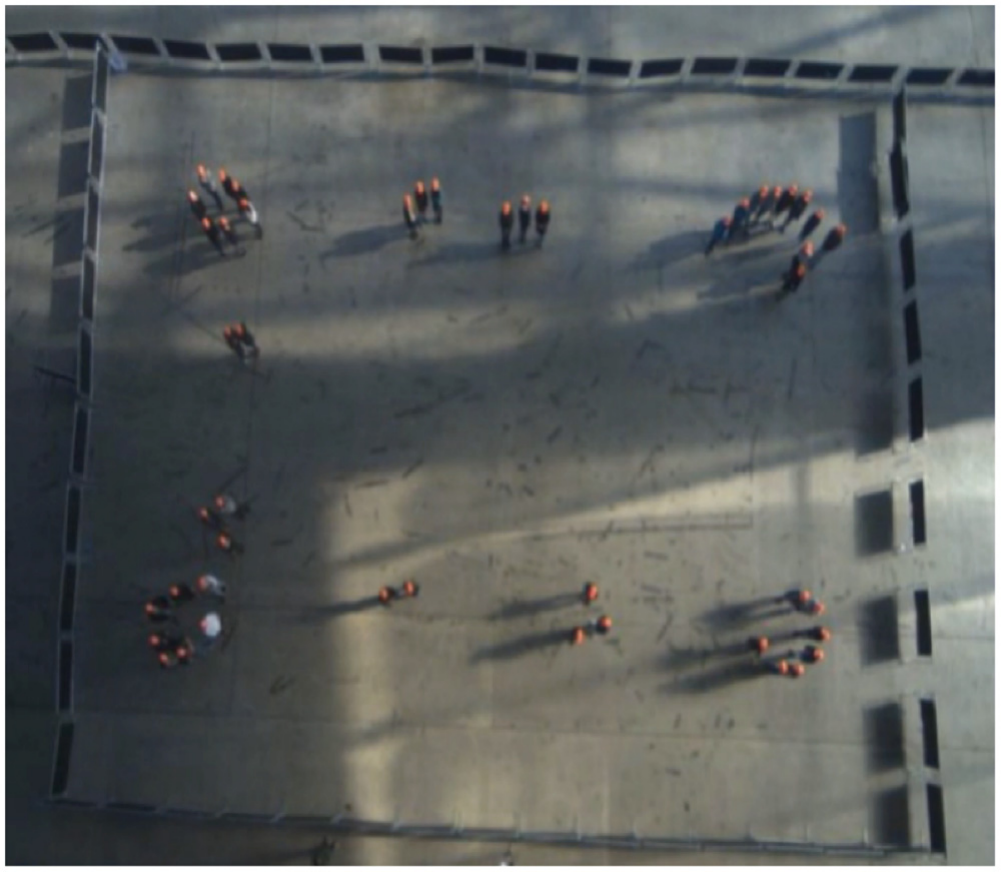
Aerial view of participants. Aerial view of participants during the group formation task, from which proximity and movement data were extracted.

After providing informed consent (including consent to appear anonymously on video), participants were assigned a participant number, which they wore on an orange baseball cap (orange improved the contrast of their heads against the ground, thereby minimizing tracking errors; participants were told that the cap’s purpose was to make their participant number visible). All participants were first photographed by a research assistant before being led, one-by-one in order of their participant number, into view of the video camera—a 20m × 30m space marked off by 1m high crowd control barriers (see Fig 1). Participants completed several activities over the course of the next hour, only a subset of which are reported here. First, participants were asked to “mingle while the experimenters set up the study” with the only constraint being to “stay in the study space”. Second, participants were instructed to order themselves, by participant number, around the periphery of the space, and then to “take five steps in and form groups of any size and composition,” and to raise their hands when their group was established. Once stable groups were formed, participants were asked to form new groups, from their current positions, two more times, and then to repeat the entire process twice over, creating nine total observations (i.e., three replications of three trials). Throughout this grouping process (and the experimental procedure), participants were given no additional instructions, and were neither encouraged nor discouraged from communicating.

Finally, participants were informed that, prior to the study, the experimenters had scattered 500 1-inch washers randomly around the experimental area, and that the participants would have to work together to pick them up and deposit them in a large basin at one corner of the experimental area. Participants were told that the experiment would end only when all the washers had been deposited in the basin. Participants began this task in the middle of the experimental space, and upon a signal by the experimenter, proceeded to pick up washers and deposit them, one at a time, in the basin. Once all washers were collected, participants were paid and dismissed; they were given a full debriefing by email one week later, including an option (which none took up) to have their data removed from analyses.

### Data preparation

Following data collection, we determined the x-y pixel coordinates of each participant’s head, within each video frame, for the entirety of the experiment. The tracking was done automatically using software custom-designed by Animation Research Limited (ARL), a New Zealand-based company specializing in software tracking. The software works by automatically extracting sets of image patches for each participant and finding these patches in the subsequent frames of the video sequence using computer vision techniques, such as template matching [74] and histogram-based matching [75]. (A more detailed description is available in “S1 text.”) The output of the tracking software– 30 sets of x-y coordinates per second for each participant—can be converted, via custom MATLAB routines into a number of different, analyzable parameters, such as the distance of each individual to the center of a group, the size and composition of subgroups that form, and the speed with which individuals move through the space.

The location data were subsequently linked to individuals’ demographic data, which they had provided online prior to the study. Physical attractiveness was added to the data file by collecting off-line ratings of the participants’ photographs (taken on the day of the study) from three hypothesis-blind research assistants (two females, one male). Coders’ responses showed acceptable internal consistency, Krippendorff’s α = .75, and were therefore averaged into a single attractiveness score. All analyses reported in this paper controlled where appropriate for effects of two experimental manipulations (reported elsewhere) that were conducted across sessions, but which were not relevant to the questions examined here.

## Results

**Fig 2:**
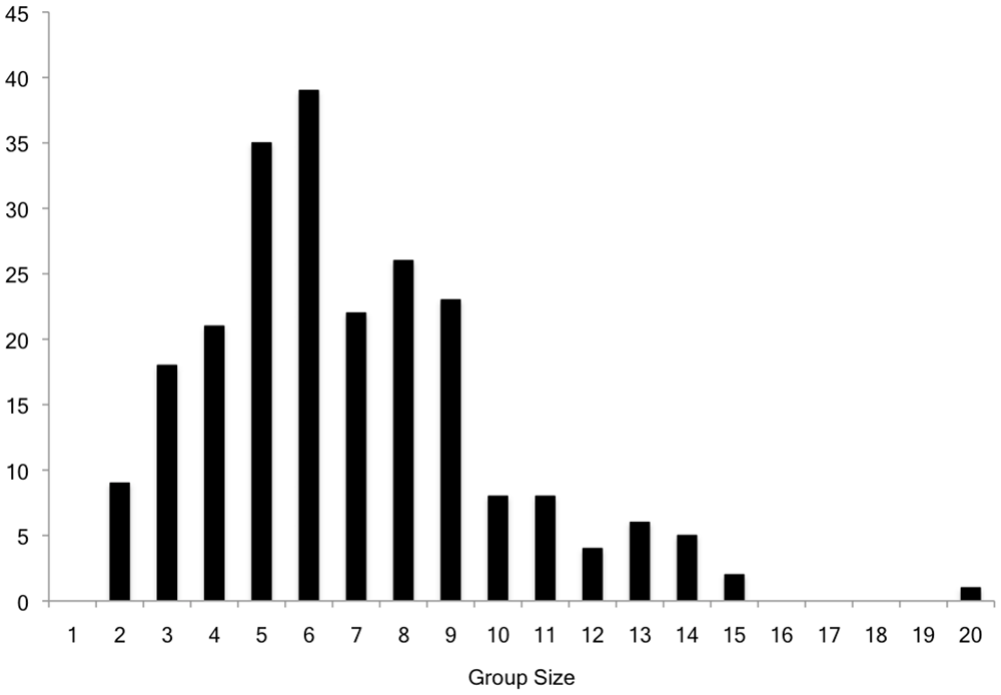
Distribution of group sizes. Frequency histogram displaying the distribution of group sizes across the 227 groups formed during the study.

Participants formed a total of 227 groups across a total of 36 trials. Group sizes were normally distributed, with a mean of 6.8 members (SD = 3.0), and a median and mode of 6; groups ranged from 2 to 20 members in size (see Fig 2). Number of groups and group size did not differ significantly across the nine trials of the study.

### Group Composition

To examine group composition, we used participants’ attractiveness and gender to predict these same traits among the other members of the groups they joined. For example, to the extent that individuals cluster by attractiveness, participants’ own attractiveness should predict the attractiveness of their groups. Thus, gender, attractiveness, and control variables (group size, participant age, and participant minority status) were entered into two repeated measures multilevel models predicting group gender and attractiveness composition respectively, with the 9 experimental trials nested within 172 participants. Group gender and group attractiveness were modeled as level 1 variables since they varied across trials, while participant gender and attractiveness were modeled as level 2 variables. Intercepts were modeled as varying across participants to account for the nested data structure. Parameters were estimated using a restricted maximum likelihood algorithm. In these models, our hypotheses regarding gender and attractiveness were partially supported: there was a non-significant gender-matching effect, *b* = .02, *t*(165) = 1.65, *p* = .10, CI [-.005, .05], and a significant attractiveness matching effect, *b* = .06, *t*(165) = 2.74, *p* = .007, CI [.02, 10]: groups were more similar in attractiveness than would be expected by chance.

In a second pair of multilevel models, we examined dynamic group composition effects by adding trial number as a level 1 predictor and modeling the cross-level interaction of trial number with participant gender and attractiveness, respectively. That is, in one model, we entered gender, trial number, and their interaction term as predictors of group gender composition (while controlling for participant attractiveness), and in a second model we entered attractiveness, trial number, and their interaction term as predictors of group attractiveness composition (while controlling for participant gender). Both models included group size, participant age, and participant minority status as control variables. In these models, the aforementioned main effects of gender and attractiveness did not change, but were qualified by a significant gender × trial interaction, *b* = -.009, *t*(1373) = -2.09, *p* = .04, CI [-.02, -.001], and a marginal attractiveness × trial interaction, *b* = -.008, *t*(1373) = -1.83, *p* = .07, CI [-.02, .001], such that both effects grew weaker with subsequent trials.

### Centrality

**Fig 3:**
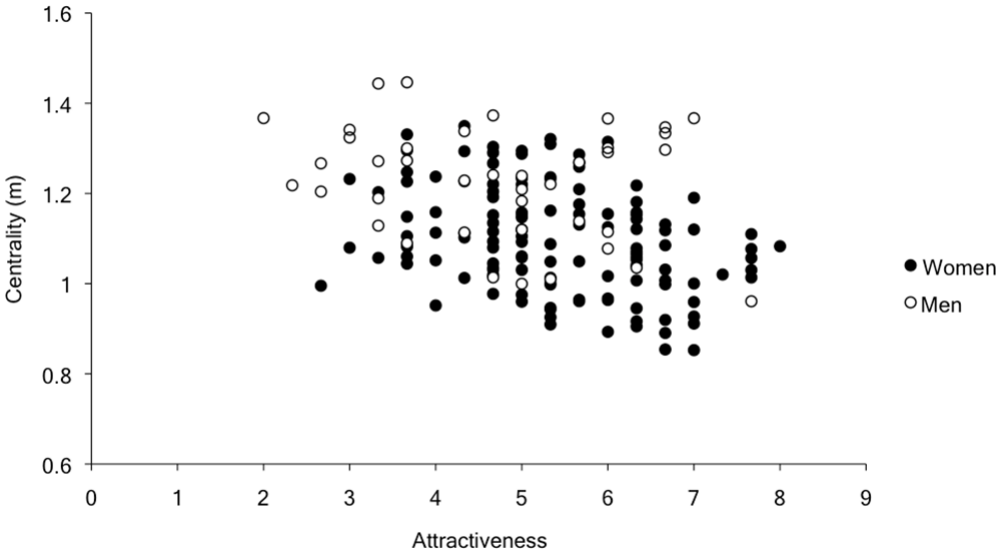
Centrality as a function of participant attractiveness and gender.

To examine the centrality of participants’ position in their groups, we calculated the mean distance between each individual and the geometric center of each group he or she joined. These distances, which were normally distributed with a mean, median, and mode of 1.1m (SD = .23m), were then analyzed in a multilevel model similarly to the composition effects. Attractiveness, age, minority status, and gender were each entered as main effect predictors. As in the composition model, group size was entered as a control variable (group size correlated *r* = .45 with the centering data). The results revealed significant independent effects of attractiveness, *b* = -.03, *t*(165) = -2.05 *p* = .04, CI [-.06, -.001], and gender, *b* = -.10, *t*(165) = -2.63 *p* = .009, CI [-.18. -.03]; more attractive people tended to be positioned closer to the center of their groups, and women were more centrally located than men (Ms = 1.10m versus 1.22m, *SD*s = .25m versus .22m). These effects are plotted in Fig 3. In a follow up model, we entered trial number and the cross-level interaction of trial number with gender, attractiveness, age, and minority status. In this second model, both the gender and attractiveness effects did not change, and there were no significant interactions with trial number.

### Social distance and social loafing

To test the relationship between social distance and cooperation, we first calculated the distance that each participant stood from all other participants in their experimental session, averaged across the entire five minute mingling phase of the study. This “mingling distance” (*M* = 8.65m, *SD* = 2.30m) was then regressed on the speed with which each individual searched for washers later in the study (*M* = .07m/s, *SD* = .04m/s), controlling for participants’ gender, attractiveness, age, and minority status. This regression was not performed in a multi-level analysis, since both mingling and foraging occurred in single trials. Results revealed a significant positive effect, *β* = .25, *t*(171) = 4.14, *p* < .001, CI [.002, .006], such that the closer a participant stood to others in the experiment, the *less* effort they put into helping the group later in the study. This effect remained significant (*p* < .001) when embeddedness during the foraging task was added to the model. Embeddedness during foraging was uncorrelated with speed (*p* = .93).

## Discussion

This study represents a unique approach to studying an unknown phase of the development of social groups, the moment when unrelated individuals make the first overtures of affiliation to others. Although many social scientists, in diverse fields, have conceptualized affiliation as physical distance, none have had the methodological tools to quantify distance under macro-level, yet controlled conditions. Using a ceiling-mounted camera, state-of-the-art color matching software, and a series of original statistical scripts, we were able to calculate unobtrusively participants’ locations in space at high physical and temporal resolution. From these basic data we can compute not only basic descriptive statistics about group formation, duration, and constitution, but also study how these indices are influenced by other behavioral, individual difference, and motivational variables.

The purpose of the study was to explore just a few of these metrics, both to fill gaps in our understanding of group formation, but also to demonstrate the potential of our methodology. First, we found that groups formed around attractiveness and, to a lesser extent, gender: attractive people, and women, were more likely to join groups of other attractive people and women, respectively, although these homophily effects decreased as participants formed subsequent groups. Second, we found that attractiveness and gender were significantly related to participants’ likelihood of standing in the geometric center of these groups, such that women and attractive participants were more likely to be closer their group’s center. It is not clear in the correlational analyses we conducted whether attractive people and women became the seeds of groups of similar others—attractors in Pennebaker’s terminology—or whether they sought out the center of groups after they started to form [27], but the nature of naturally forming groups, in which individuals converge radially, suggests that attractive individuals and women were the ones being sought out, and that they did not shoulder their way into already-formed groups. Future research is needed, however, to test this claim.

Finally, we were able to predict participants’ cooperation from their embeddedness among the full participant sample as they mingled at the beginning of the study. Participants who were closer on average to other participants at the beginning of the study, were also the ones who were less cooperative at the end of it, that is, who searched the experimental space more slowly in a task that required everyone’s help. The results are consistent with the association between anonymity and social loafing—the first such demonstration in the literature—although further research is required to identify the true mechanism behind this relationship. Specifically, we cannot at the present time be certain of the motivations behind participants’ crowding and foraging styles—or, indeed, whether the two behaviors are causally related. At the same time, the fact that participants’ embeddedness *during* the foraging did not predict loafing rules out some explanations of the effect, for example that loafing participants were simply distracted by their neighbors, or that they intentionally hid themselves among other participants during the task. Rather, the most likely account at this time is that anonymity primed subsequent social loafing, although more research is needed to directly test this hypothesis. In any case, it is noteworthy that people’s “affiliative” overtures may sometimes predict behavior that is anything but prosocial.

Our study also generated descriptive data about groups that raise interesting new questions. For instance, why did groups converge around a median of 6? One possibility is that the number relates to the capacity of working memory (i.e., Miller’s “magic number” of 7 +/- 2 [76]). Two thirds of our participants joined groups between five and nine members, tempting one to draw a connection between participants’ cognitive capacities and the number of people with whom they feel comfortable affiliating. Other research also suggests that humans can represent up to five orders of intentionality, which may constrain the size of groups that form in natural contexts [77].

Of course, the “natural group size” is likely dependent on a number of contextual factors, such as the degree of ambient noise, type of conversation, cultural differences, or group composition. For example, Dunbar and colleagues’ analysis of “freely forming conversational groups” determined a “magic number” of four, which they attributed to constraints on language processing (groups greater than four are too loud and busy for conversations to continue) [78]. In the present case, groups were not given time to engage in conversation at length, and the purpose of the groups participants formed was left intentionally ambiguous. Therefore, it is likely that group size in the current study reflects anticipated cognitive demands, rather than physical limitations that group numbers impose. Furthermore, it is quite possible that, over time, and as the groups’ goals became more evident, group sizes would have evolved in light of changing social dynamics, and our methodology is ideally suited to study such evolution.

More generally, we propose that the current methodology—constructing a stadium-size laboratory and applying state-of-the-art tracking technology to participants’ social behavior within it—could be used to test major social psychological theories with a unique balance of external validity and control. Indeed, there are hundreds of theories that are well suited for in-vivo spatial tracking; the three questions we examine here are only a sample of the hypotheses that could be tested in a “stadium laboratory.” Readers may, of course, take issue with the ecological validity of our paradigm (we seldom interact with friends and family within empty stadiums). However, by definition, *no* controlled study is exactly like “real life,” so the relevant question is whether an experimental context mimics real life in theoretically relevant ways. Here, the relevant ways include participants’ ability to naturally form groups and cooperate with each other without the interference of an experimenter or the confines of a closed laboratory space. As a result, we believe that in vivo spatial tracking is an effective compromise between laboratory control and naturalistic observation, which can be used to answer longstanding, fundamental questions about group dynamics.

## Supporting Information

S1 Data (XLSX)

S1 Text (DOCX)
