# Supplementary material for: Incipient Social Groups: An Analysis via In-Vivo Behavioral Tracking
Source: PLoS One. 2016 Mar 23;11(3):e0149880. doi: 10.1371/journal.pone.0149880 (PMC4805293; doi:10.1371/journal.pone.0149880)
Supplement: S1 Text — (DOCX) [file pone.0149880.s002.docx]

Supplementary Material for “Incipient Social Groups: An analysis via in-vivo behavioral tracking (Halberstadt et al., under review at PlOSone)

**Further explanation of tracking methodology.**

There are several challenges for computer-vision-based tracking of people, and in particular for keeping track of single members of groups. The first challenge is the occurrence of dynamic backgrounds (e.g., when surveiling a busy street or when using a moving camera). In this case, perspective changes caused by angle of view, as well as occlusions, can inhibit effective tracking. Furthermore as people move and change the positions of their limbs, their appearances and shapes change over time. Another issue is the close grouping of people. When people often approach each other, groups of individuals can sometimes be mistaken for a single person.

To meet these and other related challenges we used a static, monocular camera with a fixed focal length, mounted in the roof of the stadium, looking directly down on participants. These choices minimized the problems of perspective and occlusion, and allowed us to assume a static background, making it easier, in turn, to apply background extraction methods. All participants wore orange baseball caps to further facilitate their discrimination from the background.

Following data collection, we used custom software developed by Animation Research Limited (ARL), a New Zealand-based software engineering group, to track participants in the video feed. The software supports the tracking process by automatically extracting sets of image patches for each participant and finding these patches in the subsequent images of the video sequence using computer vision methods, such as template matching and histogram-based matching.

To use the software, the user selects a tracking target of interest (i.e. a participant in the study) with a mouse click over the target’s head, which defines the template (a box around the starting point) to be matched**.** The program assumes, given the use of the static camera, that all participants are part of the dynamic foreground. In order to be robust against camera noise, we used Gaussian-mixture-model background subtraction that creates a background model of static parts of the scene and defines pixels that match the characteristics of this model as background. The result of this first step is a binary mask containing only foreground pixels. Using the extracted foreground pixels as an input mask, the program creates a subregion within a defined search space, and then performs template matching using normalized cross-correlation and returns the location of the best match between the previously stored templates and the subregion.

A well-known problem of template matching methods is “drift” created by small errors in the location of the best template match. Over time, these errors can accumulate and can store templates that are no longer connected to the object of interest (i.e., the participant). In order to avoid this problem, we added a correction using a pre-computed histogram of the initial template to compute the probability of the found image patch in relation to the distribution of color values in the initial patch using back-projection. We then used the computed probability to find the centroid of the object of interest. Thus, the template used for template matching will only be updated with the image pixels of the new corrected location if the histogram-based matching is successful.

The result of the tracking method is a 2D location in image space that describes the computed position of one tracked participant. The method can be used for multiple objects at the same time, meaning that all participants in an experiment can be tracked simultaneously. In order to obtain a smoother movement, the tracking method uses a Kalman filter to compensate for tracking errors. The result is a set of x-y coordinates for all experimental participants. In our research, we used a camera that operated at 30 frames per second, meaning that we generated 30 sets of coordinates per second for each participant.
